# Quels patients souffrants de trouble bipolaire type I font des tentatives de suicide?

**DOI:** 10.11604/pamj.2020.37.116.24787

**Published:** 2020-10-02

**Authors:** Mariem Chakroun, Yosra Zgueb, Donia Ben Khaled, Uta Ouali, Rabaa Jomli, Fethi Nacef

**Affiliations:** 1 Service de Psychiatrie «A», Hôpital Razi, la Manouba, Tunis, Tunisie

**Keywords:** Trouble bipolaire, suicide, facteurs de risque, Bipolar disorder, suicide, risk factors

## Abstract

Le suicide est un problème de santé publique retrouvé dans la plupart des maladies psychiatriques notamment dans le trouble bipolaire (TB). Le but de ce travail était d´estimer la prévalence des tentatives de suicide (TS) et de déterminer les différents facteurs qui lui sont associés au sein d´une population de patients atteints de trouble bipolaire type I (TB I). Etude transversale et descriptive auprès de 150 patients. Nous avons eu recours à une fiche de renseignements, aux questionnaires d´évaluation des tempéraments affectifs, de l´addiction à l´alcool et des addictions aux drogues. La comparaison entre suicidants (23,3%, n = 35) et non-suicidants (76,7%, n = 115) a permis de dégager, les facteurs de risque de suicide suivants: l´addiction au cannabis, l´addiction aux psychotropes, un âge avancé, un antécédent familial de TB, de dépression, de TS et de décès par suicide, des antécédents personnels somatiques, un trouble de la personnalité associé en particulier la personnalité histrionique, un tempérament dépressif, un premier épisode thymique de nature dépressive, la récurrence des épisodes thymiques et une longue durée d´évolution de la maladie. On a également identifié deux facteurs protecteurs: l´acide valproïque et un nombre plus élevé de frères et sœurs. En étude multivariée les facteurs de risque étaient: l´âge, un antécédent familial de TB, un antécédent familial de TS et l´addiction au cannabis. Une attention particulière se doit d'être portée aux déterminants associés aux comportements suicidaires chez les patients atteints de TB I afin d'adopter des stratégies préventives et thérapeutiques efficaces.

## Abstract (en)

Suicide is a public health problem affecting majority of patients with psychiatric diseases, including bipolar disorder (BD). The purpose of this study was to estimate the prevalence of suicide attempts (SAs) and to identify different factors associated with it in a population of patients with bipolar disorder type I (BD I). We conducted a cross-sectional and descriptive study of 150 patients. We used a fact sheet, questionnaires assessing affective temperaments, alcohol addiction and drug addiction. A comparison between suicidal (23,3%, n = 35) and non-suicidal (76,7%, n = 115) subjects enabled the identification of the following risk factors for suicide: cannabis addiction, addiction to psychotropic drugs, advanced age, a family history of BD, depression, SA and suicide deaths, a personal somatic history, a personality disorder associated, in particular, with histrionic personality, depressive temperament, a first thymic depressive episode, recurrent thymic episodes and long-term disease progression. Two protective factors were also identified: valproic acid and a higher number of siblings. In a multivariate study risk factors included: age, a family history of BD, a family history of SA and cannabis addiction. Particular attention should be given to determinants associated with suicidal behavior in BD I patients in order to adopt effective preventive and therapeutic strategies.

## Introduction

Le suicide est la 15^e^ cause de mortalité dans le monde avec environ un million de suicides annuels et environ 20 fois plus de tentatives de suicide (TS) [1]. On estime que 90% des victimes de suicide souffrent d'un trouble psychiatrique, principalement les troubles liés à l'usage de substances psychoactives (SPA), les troubles de la personnalité et les troubles de l'humeur, avec à leur tête le trouble bipolaire (TB) [2, 3]. Le TB est une pathologie psychiatrique fréquente, touchant un à deux pour cent de la population générale [4]. On estime que les personnes atteintes d´un TB ont un risque de suicide 20 fois plus élevé que dans la population générale [5, 6] et que la mortalité y est supérieure de 20 à 30% [7].

Dans les sociétés arabo-musulmanes, le suicide est considéré comme une atteinte au sacré et représente une des désobéissances divines les plus capitales, faisant de ce sujet un tabou. Le patient souffrant de TB et ayant déjà fait une TS, se trouvant désormais doublement stigmatisé et rejeté, est davantage susceptible de récidiver.

L´individualisation des facteurs de risques suicidaires spécifiques dès le début de la prise en charge ainsi que tout au long du suivi de chaque patient est de ce fait primordiale. Ceci-dit, très peu de travaux ont étudié les facteurs prédictifs de comportements suicidaires au sein d´une population de patients suivis pour TB dans les sociétés arabo-musulmanes.

Les objectifs de cette étude étaient de déterminer la prévalence des tentatives de suicide (TS) chez des patients souffrant de trouble bipolaire de type I (TB I), de prélever leurs différentes caractéristiques sociodémographiques, cliniques et thérapeutiques et de chercher une éventuelle association entre les caractéristiques suscitées et le passage à l´acte suicidaire.

## Méthodes

Il s´agit d´une étude descriptive et transversale qui s´est déroulée sur une période de sept mois entre le 01/10/2018 et le 01/05/2019. Elle a été menée au sein des services hospitalo-universitaires de psychiatrie « A » et « F » de l´hôpital psychiatrique « RAZI » de la Manouba. Nous avons recruté des patients ayant consulté à la postcure des services suscités chez qui le diagnostic de TB I a été retenu selon les critères diagnostiques de la cinquième version du manuel diagnostic et statistique des troubles mentaux (DSM-5) [8]. Les critères d´inclusion étaient: un âge supérieur ou égal à 18 ans, patient en phase d´euthymie attestée selon l´échelle d´anxiété et de dépression de Zigmond et Snaith (HADS) (score < 8), et l´échelle d´évaluation de la manie de Young (score < 13). Patient dont le dernier épisode thymique remonte à plus de quatre mois. Ces patients étaient consentants à participer à cette étude après avoir été informés de ses objectifs. Les critères d´exclusion étaient: la présence d´un retard mental, de troubles cognitifs majeurs, de TB induit par une substance ou secondaire à une maladie générale.

Le recueil des données sociodémographiques et des caractéristiques cliniques et thérapeutiques du trouble a été fait initialement à partir des dossiers médicaux des patients puis vérifiées et complétées lors des entretiens directs avec les patients.

L´évaluation psychométrique était faite par cinq échelles: l´échelle d´anxiété et de dépression (Anxiety And Depression Scale) dans sa version arabe (HADS) de Zigmond et Snaith [9], l´échelle d´évaluation de la manie de Young (YMRS) [10], le questionnaire d´évaluation de l´addiction à l´alcool (CAGE) [11], le questionnaire des addictions aux drogues (DUDIT) en langue arabe [12] et le questionnaire d´évaluation des tempéraments affectifs (TEMPS-A) de Memphis, Paris et San-Diego (TEMPS). Concernant le TEMPS-A, nous avons eu recours à une version validée en langue arabe [13]. Cette échelle comporte 110 items pour les femmes et 109 items pour les hommes se référant aux cinq dimensions qui évaluent le tempérament dépressif, le tempérament cyclothymique, le tempérament hyperthymique, le tempérament irritable et le tempérament anxieux. Le score factoriel de chacune des cinq dimensions se calcule en cotant un point pour chaque réponse positive par item. Le score du tempérament irritable a été multiplié par 0,95 chez les femmes pour tenir compte de la différence des nombres des items entre les deux genres [13]. Enfin, le score total de chaque dimension est le résultat de la somme de toutes les réponses affirmatives.

**Analyse statistique:** l´étude statistique a été réalisée au moyen du logiciel Statistical Package for the Social Sciences (SPSS) dans sa version 25 pour Windows. Nous avons procédé à une étude descriptive puis à une étude analytique.

**Étude descriptive:** pour les variables qualitatives, nous avons calculé des fréquences simples et des fréquences relatives (pourcentages). Pour les variables quantitatives, nous avons déterminé les moyennes, les médianes, les écarts-types [ou déviations standards] ainsi que les valeurs extrêmes. La standardisation des scores des tempéraments affectifs a été obtenue par le calcul des scores z. Le score z est la moyenne pondérée des cotes z qui sont calculés selon la formule suivante: [score du tempérament chez le sujet-score moyen de ce tempérament chez l´ensemble des sujets] divisé par l´écart type. Le concept du tempérament affectif dominant a dérivé de la comparaison des scores z de chaque sujet dans chacun des cinq tempéraments. Les scores z ont été gradués en: = -1DS; ] -1DS, 0DS [; [0DS, +1DS [; [+1DS, +2DS [; = +2DS. Les sujets ayant des scores supérieurs à plus de 2DS par rapport aux scores moyens ont été considérés comme ayant un tempérament dominant spécifique.

Pour dégager les facteurs prédictifs de TS, la population d´étude était divisée en deux groupes selon les antécédents de tentative de suicide (TS): les suicidants (les patients ayant effectué au moins une TS au cours de leur vie); les non suicidants (il comprend les patients n´ayant jamais effectué de TS). Les comparaisons de 2 moyennes sur séries indépendantes ont été effectuées au moyen du test t de Student pour séries indépendantes. Les comparaisons de plusieurs moyennes (> 2) sur séries indépendantes ont été effectuées au moyen du test ANOVA à un facteur. Les comparaisons des pourcentages sur séries indépendantes ont été effectuées au moyen du test de chi-deux de Pearson, et en cas de non-validité de ce test par le test exact bilatéral de Fisher. Le seuil de signification a été fixé à 0,05. L´étude des corrélations entre les variables quantitatives continues a été effectuée au moyen du coefficient de Spearman. Nous avons construit un modèle de régression logistique binaire hiérarchique, avec comme variable dépendante la présence ou non d´antécédent de TS, et comme variables indépendantes; bloc 1: les variables âge, sexe, antécédents familiaux de TB, antécédents familiaux de TS, antécédents personnels somatiques, trouble de la personnalité, addiction au cannabis, nature dépressive du premier épisode thymique et nombre des épisodes de nature dépressive. Ces variables ont été choisies en fonction des résultats de l´analyse univariée et en fonction des données de la littérature. A noter que pour éviter le problème de multi-colinéarité, nous avons dû choisir d´une part entre antécédents familiaux psychiatriques et antécédents familiaux de bipolarité et d´autre part entre l´addiction au cannabis et l´addiction aux psychotropes étant donné que dans ces deux cas, les variables en question étaient très liées. Dans les deux cas, nous avons choisi la variable donnant l´information clinique la plus spécifique. Nous avons procédé de la même manière pour le nombre total des épisodes thymiques et ayons opté pour les épisodes de nature dépressive. Pour faciliter l´interprétation des résultats lors de la régression, on a regroupé les premiers épisodes thymiques de nature maniaque et ceux de nature mixte; bloc 2: les scores des différents tempéraments.

## Résultats

Notre étude a inclus 150 patients suivis pour un trouble bipolaire type I. Données liées au suicide: des antécédents d´idées suicidaires ont été retrouvés chez 94 patients (62,7%), la prévalence des TS était de 23,3% (n = 35). La TS a été jugée grave dans 54,3% des cas. Seize patients (45,7%) avaient fait plus d´une TS dans leur vie. L´âge moyen lors de la première TS était de 32,06 ± 9,3 ans. Le nombre moyen des TS était de 2,66 ± 2,83. Le moyen de la TS était dans la majorité des cas un moyen non physique à type d´ingestion médicamenteuse volontaire dans 57% des cas (n = 53). Chez 88,6% des patients suicidants (n = 31), la TS était concomitante à un épisode thymique. Il s´agissait d´un épisode dépressif chez 83,9% de ces patients (n = 26) et mixte chez 16%, (n = 5).

Comparaison des données sociodémographiques entre les deux groupes: l´âge moyen des suicidants était significativement plus bas chez les non suicidants par rapport à celui des suicidants (40,96 ± 9,61 ans vs 46,51 ± 11,36 respectivement; P = 0.005). Alors que le nombre des frères et sœurs était significativement plus élevé chez les non suicidants par rapport au non suicidants (5,85 ± 2,02 vs 5,03 ± 2,08 respectivement; P = 0.039).

Comparaison des données anamnestiques: des antécédents familiaux psychiatriques étaient présents chez 68,6% des suicidants et chez 47,8% des non-suicidants. Cette différence était statistiquement significative (p = 0,031). Des antécédents personnels somatiques étaient présents chez 40% des suicidants et chez 16). Des antécédents personnels somatiques étaient présents chez 40% des suicidants et chez 16,5% des non suicidants. Cette différence est statistiquement significative (p = 0,003) (Tableau 1).

**Tableau 1:** la comparaison des données anamnestiques entre les suicidants et les non suicidants

	Avec TS^*^ N=35 [23,3%]	Sans TS^*^ N=115 [76,7%]	P
**Antécédents familiaux psychiatriques**			
Oui	24 [68,6%]	55 [47,8%]	0,031
Non	11 [31,4%]	60 [52,2%]	
**Trouble bipolaire**			
Oui	18 [51,4%]	28 [24,3%]	0,002
Non	17 [48,6%]	87 [75,7%]	
**Schizophrénie**			0,647
Oui	3 [8,6%]	13 [11,3%]	
Non	32 [91,4%]	102 [88,7%]	
**Dépression**			
Oui	7 [20%]	9 [7,8%]	0,041
Non	28 [80%]	106 [92,2%]	
**Trouble anxieux**			
Oui	0 [0%]	1 [0,9%]	1
Non	100 [100%]	114 [99,1%]	
**Antécédents familiaux de TS**			
Oui	5 [14,3%]	2 [1,7%]	0,008
Non	30 [85,7%]	113 [98,3%]	
**Antécédents familiaux de décès par suicide**			
Oui	8 [22,9%]	4 [3,5%]	0,001
Non	27 [77,1%]	111 [96,5%]	
**Antécédents personnels somatiques**			
Oui	14 [40%]	19 [16,5%]	0,003
Non	21 [60%]	96 [83,5%]	

TS^*^ : tentative

Comparaison des données cliniques: parmi les suicidants, cinq patients (14,3%) avaient un trouble anxieux comorbide et huit patients (22,9%) avaient une addiction à l´alcool, mais ces associations n´étaient pas statistiquement significatives. Par contre, le passage à l´acte suicidaire était significativement associé à l´addiction au cannabis (p = 0.013). Un trouble de la personnalité, représenté essentiellement par la personnalité histrionique était également significativement présent chez les suicidants (p = 0,011). Le score moyen du tempérament hyperthymique chez les suicidants était le score le plus élevé mais seul le tempérament dépressif qu´était significativement associé au passage à l´acte suicidaire (p = 0,048) Tableau 2.

**Tableau 2:** la répartition de la population selon les comorbidités psychiatriques et la nature du tempérament

	Avec TS^*^ N=35 [23,3%]	Sans TS^*^ N=115 [76,7%]	P
**Trouble anxieux co-morbide**			
Oui	5 [14,3%]	21 [18,3%]	0,586
Non	30 [85,7%]	94 [81,7%]	
**Trouble de la personnalité**			
Oui	9 [25,7%]	9 [7,8%]	0,014
Non	26 [74,3%]	106 [92,2%]	
**Personnalité histrionique**			
Oui	6 [17,1%]	4 [3,5%]	0,011
Non	29 [82,9%]	111 [96,5%]	
**Personnalité antisociale**			
Oui	2 [5,7%]	4 [3,5%]	0,624
Non	33 [94,3%]	111 [96,5%]	
**Personnalité limite**			
Oui	0 [0%]	1 [0,9%]	1
Non	100 [100%]	114 [99,1%]	
**Personnalité dépendante**			
Oui	0 [0%]	1 [0,9%]	1
Non	100 [100%]	114 [99,1%]	
**Addiction à l´alcool**			
Oui	8 [22,9%]	18 [15,7%]	0,319
Non	27 [77,1%]	97 [84,3%]	
**Addiction au cannabis**			
Oui	7 [22%]	6 [5,2%]	0,013
Non	28 [80%]	109 [94,8%]	
**Addiction aux psychotropes**			
Oui	5 [14,3%]	5 [4,3%]	0.05
Non	30 [85,7%]	110 [95,7%]	
**Scores moyens des tempéraments [TEMPS-A]**			
**Tempérament dépressif**	10,8±3,72	9,48±3,35	0,048
**Tempérament cyclothymique**	10,6±5,68	10,26±5,67	0,757
**Tempérament hyperthymique**	11,23±5,04	12,04±4,79	0,385
**Tempérament irritable**	5,37±3,9	5,7±4,66	0,702
**Tempérament anxieux**	10,86±6,03	10,05±3,32	0,506

TS^*^ : tentative de suicide

Un premier épisode de nature dépressive était retrouvé chez 54,29% des suicidants. Cette différence était statistiquement significative (p = 0,031). Plus le nombre d´épisodes thymiques était important plus le risque suicidaire était accru. Cette association était statistiquement significative lorsqu´il s´agissait d´un épisode de nature dépressive (p = 0003). Plus le nombre d´années d´évolution était important plus le risque suicidaire était également élevé (p = 0008). Quant au traitement, l´acide valproïque était prescrit chez 65,7% des suicidants et chez 84,3% des non-suicidants. Cette différence était statistiquement significative (p = 0,016). Par contre, nous n´avons pas trouvé d´association statistiquement significative entre l´observance thérapeutique et la présence d´un antécédent de TS (Tableau 3).

**Tableau 3:** la comparaison des données évolutives et thérapeutiques entre les suicidants et les non suicidants

	Avec TS^*^ N=35 [23,3%]	Sans TS^*^ N=115 [76,7%]	P
**Âge de début du trouble [ans]**	26,46 ± 8,638	26,17 ± 0,715	0,848
**Duré du trouble non traité [mois]**	21,15 ± 38,35	10,33 ± 15,83	0,112
**La nature du premier épisode**			
Dépressif	19 [54,29%]	35 [30,43%]	
Maniaque	11 [31,43%]	61 [53,05%]	0.031
Mixte	5 [14,28%]	19 [16,52%]	
**Nombre moyen des épisodes thymiques**			
Dépressif	3,09 ± 1,93	1,53 ± 2,87	0,003
Maniaque	4,43 ± 4,15	3,99 ± 3,975	
Mixte	1,51 ± 1,65	1,23 ± 1,89	
Total	9,03 ± 4,119	6,76 ± 5,624	0,028
**Cycles rapides**			
Oui	6 [17,1%]	27[23,5%]	0,428
Non	29 [82,9%]	88 [76,5%]	
**Caractère saisonnier**			
Oui	8 [22,9%]	24 [20,9%]	0,802
Non	27 [77,1%]	91 [79,1%]	
Nombre d´années d´évolution	19,69 ± 11,295	14,82 ± 8,765	0,008
**Traitement thymoréulateur**			
**Acide Valproïque**			
Oui	23 [65,7%]	97 [84,3%]	0,016
Non	12 [34,3%]	18 [15,7%]	
**Antipsychotique**			
Oui	28 [80%]	73 [63,5%]	0,068
Non	7 [20%]	42 [36,5%]	
**Antipsychotique utilisé**			
Classique	18 [64,3%]	47[64,4%]	0,993
Atypique	10 [35,7%	26 [35,6%]	
**Benzodiazépine**			
Oui	23 [65,7%]	63 [54,8%]	
Non	12 [34,3%]	52 [45,2%]	0,252
**Benzodiazépine utilisé**			
Lorazépam	22[95,7%]	60[95,2%]	1
Diazépam + Clonazépam	1 [4,3%]	3[4,8%]	
**Antidépresseur**			
Oui	0 [0%]	12 [100%]	1
Non	35 [100%]	0 [0%]	
**Observance thépapeutique**			
Bonne	13 [37,1%]	54 [47%]	
Moyenne	12 [34,3%]	41 [35,7%]	0.321
Mauvaise	10 [28,6%]	20 [17,4%]	
Nombre moyen d´arrêts de traitement	1,6 ± 1,666	1,08 ± 1,377	0,098

TS^*^: Tentative de suicide

**Etude multivariée:** les facteurs prédictifs de TS indépendants dégagés de l´étude multivariée sont présentés dans le Tableau 4.

**Tableau 4:** facteurs de risque de TS chez les patients atteints de TB I en analyse multivariée

	P	Odds Ratio ajusté	Intervalle de confiance
Âge	0,003	0,923	0,87-0,97
Antécédent familial de TB^*^	0,002	5,504	1,914-15,828
Antécédent familial de TS^**^	0,028	14,553	1,331-159,092
Addiction au cannabis chez les hommes	0,003	11,761	2,357-58,679

* TB: Trouble bipolaire, ^**^ TS: Tentative de suicide

## Discussion

La prévalence des TS dans notre étude était de 23,3%. Ce taux est légèrement inférieur à celui retrouvé dans de nombreuses études [14, 15]. Ceci pourrait être expliqué par la place qu´occupe le suicide dans notre culture arabo-musulmane. De ce fait, les TS pourraient ne pas être systématiquement rapportées. Dans notre étude, dans presque 90% des cas, la TS était concomitante à un épisode thymique. Il s´agissait d´un épisode dépressif chez 83,9% des patients et mixte chez 16,1% des patients. Ce résultat rejoint ceux de la littérature. Dans l´étude de Jylhä *et al*. menée en 2016 en Finlande, le passage à l´acte suicidaire était concomitant à un épisode thymique dans presque la totalité des cas. Cet épisode thymique était soit un épisode dépressif majeur soit un état mixte qui sont, respectivement, 25 et 65 fois plus pourvoyeurs d´une TS qu´un état d´euthymie [16].

Dans une méta-analyse de Plans *et al*. [17], faite en 2018 et portant sur 2806 articles scientifiques, les hommes souffrant de TB I font deux fois plus de suicides aboutis que les femmes. Quant aux TS, la répartition selon le genre différait d´une étude à l´autre et d´un pays à l´autre [18, 19]. Une prévalence des TS plus élevée chez les femmes pourrait être imputée aux particularités des sociétés étudiées et à leurs caractéristiques culturelles. Prenons l´exemple des sociétés patriarcales où les femmes, ne jouissant pas d´un minimum de droits et d´émancipation, sont plus sujettes à commettre des suicides. Cette association n´a pas été retrouvée dans notre étude alors que dans la population générale, les femmes font deux fois plus de TS [20].

Le groupe des suicidants avait un âge plus avancé que celui des non-suicidants. Un âge avancé au moment de l´étude est positivement associé à un antécédent de TS. Cette association s´explique par le fait qu´un nombre d´années d´évolution de la maladie plus élevée a été retrouvé chez le groupe des suicidants.

Les conditions socioéconomiques (CSE) ne semblent pas influencer le risque de passage à l´acte suicidaire dans notre étude paradoxalement à ce que l´on trouve dans la littérature [21, 22] où les difficultés financières et les CSE défavorables constitueraient un facteur de risque de passage à l´acte suicidaire chez les patients souffrant de TB I. Des études faites par Goffin *et al*. [21] en 2016, Shabani *et al*. [23] en 2013 ou encore Bellivier *et al*. [22] en 2011 avaient toutes identifié le chômage comme étant un facteur de risque suicidaire chez les patients atteints de TB I. Vu son taux très élevé en Tunisie, et le modèle sociétal de vie en famille jusqu´à un âge avancé, le chômage n´est pas aussi stigmatisé que dans les pays occidentaux. Ceci pourrait expliquer la différence de nos résultats avec ceux de la littérature.

Il y avait une différence statistiquement significative entre suicidants et non-suicidants par rapport à la présence d´antécédents familiaux de TB I, d´une histoire familiale de TS et de décès par suicide. Ces résultats sont concordants avec ceux de la littérature où les antécédents familiaux de bipolarité et de comportements suicidaires constituent un facteur prédictif de passage à l´acte suicidaire [24, 25] surtout lorsqu´il s´agit de parenté au premier degré. Cette constatation évoque une vulnérabilité génétique au suicide [26] et souligne l´intérêt porté sur l´importance des facteurs génétiques et épigénétiques dans la suicidalité. Par ailleurs, les facteurs environnementaux sont également évoqués face à de tels résultats comme le suggère l´étude de Miklowitz et Chang [27] qui ont découvert une association entre le fonctionnement de la famille et la suicidalité. Une histoire familiale de TS ou de décès par suicide aurait donc un rôle suicidogène et une telle expérience influencerait le vécu des patients et leur perception de cet acte. Un antécédent personnel somatique a été identifié comme facteur de risque de suicide en analyse univariée. Dans la littérature, une revue systématique concernant une méta-analyse faite en 2019 affirme que les troubles somatiques sévères jouent un rôle dans le développement de la vulnérabilité et constituent un facteur de risque de suicide [17].

Concernant les comorbidités: les personnalités pathologiques ont été identifiées comme facteur prédictif de conduite suicidaire, particulièrement lorsqu´il s´agissait d´une personnalité histrionique, comme retrouvé dans la plupart des études à travers le monde [28, 29]. Concernant la personnalité borderline et la personnalité antisociale, aucune association n´a été retrouvée. Ce résultat ne rejoint pas celui de la littérature où il a été communément admis que le risque suicidaire est plus important pour des personnalités marquées par des traits d´impulsivité. Prenons l´exemple de la personnalité antisociale, qui, de par son grand risque d´inobservance thérapeutique, est sujette à développer des comportements suicidaires [30]. Les conduites addictives étaient essentiellement représentées par l´addiction à l´alcool (17%) suivies par celle au cannabis (8,7%) et aux psychotropes (6,7%). Les taux retrouvés dans notre étude sont inférieurs à ceux de la littérature. Dans une étude faite par Yerevanian et Choi aux États-Unis [31], le taux d´addiction chez des patients bipolaires s´élève à 48,5% pour l´alcool et à 36% pour le cannabis. Ces taux étaient assez similaires à ceux de Regier *et al*. [32] (46,2% pour l´alcool et 40,7% pour la toxicomanie). Une sous-estimation de la fréquence des conduites addictives par rapport à la littérature pourrait être expliquée par la peur du jugement que ressentent les patients de notre étude qui sont issus majoritairement d´un milieu rural conservateur. Ceci pourrait aussi être dû à des facteurs économiques: le coût relativement élevé de ces substances limiterait leur consommation. S´ajoutent à cela l´interdiction de la consommation de l´alcool dans la religion musulmane, et l´illégalité de la consommation récréative de cannabis dans la loi tunisienne. Quant à l´addiction à l´alcool n´avait pas d´association statistiquement significative à un antécédent de TS, tandis que l´addiction au cannabis et l´addiction aux psychotropes lui étaient hautement associées chez les hommes. Selon certains auteurs, la toxicomanie était associée au risque suicidaire chez les patients atteints de TB I mais pas chez ceux atteints de trouble bipolaire type II (TB II).

Par rapport au tempérament, en analyse univariée, le tempérament dépressif était associé d´une manière statistiquement significative avec un antécédent de TS. Un échantillon plus grand pourrait en effet démontrer un lien existant entre le tempérament dépressif et un antécédent de TS. Dans la littérature, une évaluation systématique des tempéraments chez des patients ayant fait une TS versus un groupe de non-suicidants, révèle la prédominance des tempéraments cyclothymique, dépressif, anxieux et irritable [33]. Vázquez *et al*. ont observé que le tempérament hyperthymique est protecteur contre le risque suicidaire [34].

Dans l´étude des données cliniques et évolutives: un premier épisode de nature dépressive était associé dans notre étude à un risque accru de passage à l´acte suicidaire. Ce même résultat a été fréquemment retrouvé dans la littérature comme en témoignent les études de Goffin *et al*. en 2016 [21] et celle de Cremaschi *et al*. en 2017 [25]. Dans notre étude, plus le nombre des épisodes thymiques était important plus le risque suicidaire était élevé. Cette association était statistiquement significative lorsqu´il s´agissait d´un épisode de nature dépressive. Les suicidants font plus de rechutes et d´exacerbations thymiques et seraient probablement atteints de formes plus sévères de TB. Ce résultat est semblable à celui de la quasi-totalité des études [18, 35].

Dans une étude de la cartographie de l´humeur de Judd *et al*. [36], menée sur une période supérieure à 12 ans aux USA, les patients atteints de TB I avaient une humeur déprimée pendant en moyenne 46% de leurs journées. En partant du principe que le risque de suicide est plus élevé en phase dépressive de la maladie, les patients souffrant de TB I passent une grande partie de leur temps dans une condition à haut risque suicidaire. Parallèlement, dans notre étude, nous n´avons pas trouvé d´association statistiquement significative entre risque suicidaire et présence de symptômes psychotiques lors des épisodes thymiques. Ce résultat concordait avec ceux de la littérature [35].

Concernant l´aspect thérapeutique: chez nos patients l´acide valproïque était prescrit chez 65,7% des suicidants et chez 84,3% des non-suicidants. Cette différence était statistiquement significative et témoigne du rôle protecteur de l´acide valproïque contre les TS. Ce constat a été retrouvé dans la littérature. En effet, lors d´une étude ayant pour objectif de déterminer le rôle des anticonvulsivants dans la prévention du suicide dans le TB [37], l´arrêt de l´acide valproïque a considérablement élevé le risque de comportement suicidaire, témoignant ainsi de son potentiel rôle protecteur. Par ailleurs, chez les patients prenants des AP (67%), il s´agissait de neuroleptiques classiques (NLPC) dans 64% des cas. Il n´y avait pas d´association statiquement significative entre la prescription d´un AP dans le traitement ni son type lorsqu´il était prescrit, et un antécédent de TS. Ce résultat rejoint celui trouvé par Vieta *et al*. [38] dans une étude prospective menée à Barcelone, où ils ont constaté que la rispéridone n´avait pas eu d´impact sur le risque suicidaire.

Perspectives de prévention: établir un profilage des patients bipolaires à haut risque suicidaire renforce l´action thérapeutique et l´ampleur de la prévention du passage à l´acte suicidaire. Il est important de souligner qu´on ne pourrait pas contourner tous les facteurs de risque suicidaires chez un patient donné parce que certains de ces facteurs sont tout simplement non modifiables comme l´âge, le tempérament, les antécédents familiaux psychiatriques, la nature du premier épisode thymique, et le nombre d´années d´évolution. Ils définissent un terrain de vulnérabilité bien précis. La principale action préventive serait de ce fait axée sur la prise en charge des comorbidités addictives, psychiatriques et somatiques et sur la réduction du nombre d´épisodes thymiques, à travers l´amélioration de l´observance thérapeutique. Les données concernant le rôle des comorbidités organiques sur la majoration de la vulnérabilité suicidaire soulèvent la nécessité des interventions visant à promouvoir des modes de vie sains et à effectuer des dépistages et des soins médicaux [39]. Les programmes de psychoéducation du TB ayant pour but de faire comprendre aux patients leur trouble et ses caractéristiques, leur traitement et leurs difficultés en élaborant un plan spécifique et personnalisé ont montré leur efficacité [40]. Des programmes de thérapie cognitivo-comportementale (TCC) ont été conçus pour les patients suicidaires. Ils ont également montré leur efficacité en proposant au patient un plan d´action en urgence ainsi qu´un travail cognitif approfondi à long terme [41]. Les TCC ont montré leur efficacité aussi bien dans la phase dépressive du TB ou le risque suicidaire est accru, que dans la phase d´euthymie, permettant ainsi d´agir aussi sur les comorbidités addictives et contre les actes d´automutilation présents en cas d´impulsivité [38].

## Conclusion

Le risque suicidaire a été associé à un certain nombre de facteurs: une histoire familiale de pathologie psychiatrique en particulier le TB, des antécédents familiaux de TS et de décès par suicide, des antécédents personnels somatiques, une personnalité histrionique, un tempérament dépressif, la récurrence des épisodes thymiques surtout ceux de nature dépressive, une longue durée d´évolution de la maladie et un abus de cannabis et de psychotropes. Un rôle protecteur de l´acide valproïque contre le suicide a également été mis en évidence. En effet, au-delà du diagnostic de bipolarité, c´est le cumul de plusieurs facteurs de risque qui influence le risque suicidaire. La coexistence de plusieurs facteurs identifie un sous-groupe particulier de patients souffrant de TB I dont le profil a pu être dressé au terme de cette étude. En effet, un tel profil a souvent été étudié dans les sociétés occidentales. Le point fort de notre étude, c´est qu´elle s´est intéressée au suicide, sujet tabou par excellence, dans une société arabo-musulmane ou le suicide reste un acte traditionnellement condamné par les doctrines religieuses.
